# *In Vitro* Antibacterial and Antioxidant Activities of Roasted and Green Coffee Beans Originating from Different Regions of Ethiopia

**DOI:** 10.1155/2020/8490492

**Published:** 2020-08-25

**Authors:** Tsedale Tasew, Yalemtsehay Mekonnen, Tegenu Gelana, Mesfin Redi-Abshiro, Bhagwan Singh Chandravanshi, Estifanos Ele, Ahmed Mustefa Mohammed, Hassen Mamo

**Affiliations:** ^1^Department of Biology, Kotebe Metropolitan University, Addis Ababa, Ethiopia; ^2^Department of Microbial, Cellular and Molecular Biology, College of Natural and Computational Sciences, Addis Ababa University, PO Box 1176, Addis Ababa, Ethiopia; ^3^Department of Zoological Sciences, College of Natural and Computational Sciences, Addis Ababa University, PO Box 1176, Addis Ababa, Ethiopia; ^4^Department of Chemistry, College of Natural and Computational Sciences, Addis Ababa University, PO Box 1176, Addis Ababa, Ethiopia

## Abstract

Coffee is among the most traded commodities and consumed beverages worldwide primarily for its stimulating effects. Moreover, coffee is known to contain various bioactive compounds with significant health benefits including antibacterial and antioxidant activities. However, Ethiopia as the origin of coffee and producer and exporter of varieties of *Coffea arabica* has made little study on the health aspects of this beverage. The aim of this study was to examine the antibacterial and antioxidant activities and content of coffee samples from different localities of Yorgacheffe and Jimma; and roasted, ground, and packed samples purchased from a coffee shop in Addis Ababa. Medium-roasted-boiled and lyophilized coffee extracts were tested on eight gram-negative and gram-positive bacterial strains. The agar-well diffusion method was used to test the extracts determining the minimum inhibitory and bactericidal concentrations. For coffee antioxidant activity and content, light-roasted (both field and shop samples) and green coffee bean extracts were tested using the free radical 2.2-diphenyl-l-pict1hydrazyl (DPPH) IC50 percent inhibition protocol. The samples showed strong antibacterial and antioxidant activity and substantial antioxidant content. Significant variation was noted in the antibacterial activities of the different coffee samples. Moreover, the growth-inhibitory strength of each coffee sample was variable for different test bacteria. A coffee sample with the best antibacterial activity had also the highest antioxidant activity/content. The shop coffee had the lowest bioactivity. The observed variations in the antibacterial and antioxidant activities among the samples probably indicate the diversity of the Ethiopian coffee related, among other factors, to the coffee plant genetics and agroecology.

## 1. Introduction

Coffee (*Coffea* L.), which is the most traded commodity next to oil [1], is the favorite beverage consumed worldwide and is used as an additive to a number of other beverages and foodstuffs. The two common coffee species are *Coffea arabica* and *C. robusta/canephora*. Arabica coffee, which is believed to be first known or used in Ethiopia [2], is the most common and preferred coffee type globally.

Apart from being a favorite drink mainly for its stimulating and refreshing effects, nowadays coffee is receiving augmented attention for its possible health benefits as well. It is claimed that coffee possesses an array of medicinal activities against noninfectious as well as infectious diseases. In a large longitudinal study, it was found that depression risk decreases with increasing caffeinated coffee consumption [3]. Coffee intake is associated with reduced risk of type 2 diabetes mellitus [4–6]. Further, moderate coffee consumption is inversely associated with risk of heart failure [7]. Chronic coffee consumption is associated with improved endothelial function in elderly subjects, providing a connection between nutrition and vascular health [8]. Moreover, evidence lends support to the presence of an ingredient in coffee that protects against cirrhosis, especially alcoholic cirrhosis [9]. The beneficial effects of coffee concerning noninfectious disorders are attributable to coffee's antioxidant properties.

About coffee's role against infectious agents, both the extracts of regular and decaffeinated coffee showed an inhibitory effect on hepatitis B virus replication *in vivo* and *in vitro* models [10]. Similarly, coffee extracts showed strong antibacterial activity in certain studies elsewhere [11]. However, both the antioxidant and antibacterial activities of coffee are subject to species, roasting and its degree, brewing procedure, and decaffeination [12]. Coffee varieties from different origins differ substantially according to their components. Multiple agrogeographic conditions of the coffee plant including soil type, altitude, and harvest season as well as pre-and postharvest management practices also affect the bioactivity of coffee beans.

In Ethiopia, little information is available on the antibacterial and antioxidant activity/content of the Ethiopian coffee despite the nation being the homeland and center of genetic diversity of the plant. Currently, Ethiopia is the first African and the fifth largest global producer of *C. arabica*. Further, Ethiopia produces different coffee varieties from different regional origins including wild types. This study was aimed at investigating the antibacterial and antioxidant activity/content of crude extracts of field coffee beans originating from two coffee-growing regions as well as ready-to-use shop-coffee powder.

## 2. Materials and Methods

### 2.1. Samples

In total, six green coffee bean samples (1 kg each) were obtained from different localities (farms) of two coffee-growing regions of Ethiopia. The two regions are Yorgacheffe (Y) in the south and Jimma (J) in the southwest. J is one of several coffee-growing regions in Ethiopia. According to the local Agricultural Office, J has a total area of 1,093,268 hectares, of which about 105,140 hectares was occupied by coffee plantation. The coffee farms are divided into three—small-scale farmers' holdings, giant private-owned plantations, and state farms. Coffee is the major cash crop of J. About eight districts in J have favorable climatic conditions for the coffee plant. There are varieties of local coffee types in J and the area has long history of coffee plant domestication and production. Similarly, Y has favorable agroecological environment for coffee production and it is well-known for its specialty coffee in the international market.

Three coffee bean samples from three different farms from Y were labeled Y1, Y2, and Y3. Those from J were similarly designated J1, J2, and J3. Both regions were visited between December 2017 and March 2018 acquiring the samples from the same harvest season. The altitudes where the coffee plants grew fall between 1515 and 2163 meters above sea level.

In both areas, there are dedicated coffee farmers. The farmers (named “model farmers”) who produce a special coffee under close supervision of local experts on the purpose of exportation to the international market kindly donated the samples. All the green coffee samples were obtained from ripped coffee cherries and processed by sundry. The green coffee bean samples were kept in sealed paper bags; transported to the Department of Microbial, Cellular and Molecular Biology (Biomedical Sciences Laboratory), College of Natural and Computational Sciences, Addis Ababa University; and stored under room temperature conditions until used for extraction.

Additionally, three roasted-ground-packed coffee samples (250 g each) were purchased from a local coffeehouse (company) in Addis Ababa. These samples, hereafter, are referred to as shop coffee (T) and symbolized T1, T2, and T3. The levels of roasting were different for these three samples. While T1 was light, T2 and T3 were considered medium and dark, respectively, as declared by the sellers. The coffee company established in the early 1950s is among the most preferable roasted coffee powder suppliers and coffee brewers in Addis Ababa. In general, it was claimed that T is the best quality in Ethiopia. The company states that its coffee is prepared and packed by blending the most preferable Ethiopian coffee varieties for their test, aroma, flavor, and other desirable complex features.

### 2.2. Roasting, Extraction, and Brewing

Half of the field samples were roasted (roughly light and medium) in Ethiopian traditional coffee-roasting apparatus, ground using an electric grinder, and sieved with a 0.5 mm particle size sieve. The other half was reserved for raw extraction. The medium-roasted-powdered coffee samples, as well as T, were boiled using 25 g powder in 250 ml distilled water (100 mg/ml) for 15 minutes in a flask covered with aluminum foil. The extracts were double-filtered with cotton followed by Whatman No. 1 filter paper. The filtrates were put in a deep freeze for 48 hours and lyophilized. These boiled and freeze-dried samples were stored in a refrigerator until used for testing.

The green coffee samples were ground and extracted using methanol. Similarly, the light-roasted powder was extracted with methanol after sieving. Twenty-five-gram powder of each sample was dissolved in 250 ml methanol and allowed to shake for 72 hours in a shaker (GFL, Model 3020, Germany) at 27°C and filtered with Whatman No. 1 filter paper. The methanol evaporated using a rotary evaporator (BUCHI, Germany) at 45°C followed by oven drying to remove the remaining solvent and kept in a refrigerator until used for antioxidant test.

Prior to the actual antibacterial activity test, preliminary screening tests were done using different extraction solvents (cold-water, ethyl acetate, methanol, and boiled-lyophilized water extracts; instant coffee filtrate; instant coffee lyophilized extracts; and the boiled coffee lyophilized water extract reextracted with methanol and ethyl acetate). The roasted-boiled-lyophilized coffee extracts were qualitatively screened for the most common phytochemicals known to have antibacterial and antioxidant activities following established preliminary tests.

### 2.3. Antibacterial Activity Test

A crude extract solution of the roasted-boiled-lyophilized coffee samples was prepared following a standard antimicrobial agent preparation procedure [13]. Briefly, 0.5 g of each sample extract was dissolved in 1 ml distilled sterile water (500 mg/ml) and two other twofold lower concentrations (250 and 125 mg/ml) were prepared. Chloramphenicol (0.75 mg) and distilled sterile water were used as positive and negative controls, respectively.

Standard (American Type Culture Collection (ATCC)) and clinical strains of *Escherichia coli*, *Pseudomonas aeruginosa*, *Staphylococcus aureus*, and *Salmonella typhimurium* were used in the experiment. The clinical strains were ATCC 25922, ATCC 27853, ATCC 25923, and ATCC 13311, respectively. The Ethiopian Public Health Institute kindly provided both strains of each bacterium. Each species was characterized biochemically prior to the antibacterial test. Inoculants were prepared following the direct colony suspension preparation method [13]. Individually grown colonies onto a selective medium of a particular bacterial type (*E. coli*-eosin methylene blue agar, *S. typhimurium*-Shigella-Salmonella (SS) agar, *S. aureus*-mannitol salt agar, and *P. aeruginosa*-Pseudomonas agar) were transferred onto a nutrient agar plate (streak plate) and incubated for 24 hours. Fresh suspensions were made using pure bacterial colonies picked up by sterile inoculating loop and dissolved in sterile physiological saline solution. The concentration of the bacterial suspension was then adjusted with a turbidity visually comparable to 0.5 McFarland standard prepared by mixing 0.5 ml of 1.75% (*w*/*v*) barium chloride dihydrate (BaCl_2_.2H_2_O) with 99.5 ml of 1% (*v*/*v*) sulfuric acid (H_2_SO_4_), which is equivalent to approximately 1 − 2 × 10^8^ colony-forming units per ml (CFU/ml).

Antimicrobial activity was determined using the agar-well diffusion method [14]. Five wells with a six-millimeter (mm) diameter size were punched on each Mueller-Hinton agar (MHA) plate using a sterile cork borer. Bacterial suspensions of each strain were prepared with concentrations adjusted to a 0.5 McFarland standard. Of each suspension, a sterilized cotton tip was soaked and spread on MHA plates. Three wells on each plate were filled with 40 *μ*l of coffee extract solutions with different concentrations (500 mg/ml, 250 mg/ml, and 125 mg/ml); central wells of each plate were filled with chloramphenicol, the powerful broad-spectrum antibiotic generally considered active against both gram-positive and gram-negative bacteria. The other single well was filled with the negative control. After incubation for 24 hours at 37°C, the diameter of the inhibition zones (including the diameter of the well) was measured in three different directions in order to minimize error. The measurement was expressed as mm of inhibition against the test bacteria. The test was done thrice and the mean measurements recorded.

### 2.4. Minimum Inhibitory and Bactericidal Concentrations

Following the triplicate antibacterial tests up to the concentration of 125 mg/ml for each sample type, the minimum inhibitory concentration (MIC) was determined. 0.5 McFarland bacterial suspension was diluted into 1 : 150 by transferring 1 ml of 0.5 McFarland suspension into 150 ml of sterile Mueller-Hinton broth (MHB) by the macrodilution broth method [13] to have about 5 × 10^6^ CFU/ml, and the subsequent 1 : 2 dilution with the extracts solution brings the final inoculum to 5 × 10^5^ CFU/ml. The detail of the dilution procedure was that each 0.5 McFarland comparable bacterial suspension was transferred to separate 250 ml sterile nutrient broth container and mixed to bring 5 × 10^6^ CFU/ml. Then, 1 ml of the suspension was transferred to each of the twofold broth dilution test tubes labeled 62.5, 31.25, 15.625, 7.8125, 3.906, 1.953 and 0.976 mg/ml. A positive control contains 1 ml of the respective bacterial suspension, and in another test tube, the same volume of sterile broth was added as negative control. A single twofold dilution test was done for all the samples. Triplicate tests were performed only for the samples with the best inhibition measure, on average, against all the test bacteria.

One milliliter of the crude extract solution was transferred to the first test tube (the highest concentration, 62.5 mg/ml) and shaken well, and 1 ml from the first test tube was transferred to the next lower concentration level with shaking and went onto the final twofold serial dilution tube (0.976 mg/ml). Finally, from the last test tube, 1 ml solution was discarded to bring the equal volume. The test tubes were then incubated at 37°C for 24 hours, and the smallest concentration on which growth was inhibited was considered the MIC of the extract. The test was repeated thrice on three different days. The minimum bactericidal concentration (MBC) was determined by transferring inoculants starting from the MIC tube to the next higher concentrations onto fresh sterile nutrient agar plates using inoculating loop streak plating.

### 2.5. Antioxidant Content and Activity

To determine the antioxidant content (AC), 50 *μ*g sample and 40 *μ*g 2.2-diphenyl-l-pict1hydrazyl (DPPH) (Aldrich) were separately dissolved in 1 ml 99% methanol. Then, 4 ml of the DPPH solution was added to 1 ml of each of the coffee extract solutions and kept in the dark for 30 minutes at room temperature and absorbance read using a spectrophotometer (Christ, Germany) at 517 nm. One milliliter methanol added to 4 ml DPPH was used as a blank; ascorbic acid was the reference.

To develop the standard curve, standard ascorbic acid (7.5 mg in 50 ml methanol) was diluted to six different concentrations (3, 6, 12, 18, 24, and 30) in *μ*g/ml. Then, 1 ml of each mixed with 4 ml DPPH (40 *μ*g/ml) was incubated in the dark for 30 minutes and absorbance read at 517 nm. The AC was calculated [15] using ascorbic acid equivalent antioxidant content (50 *μ*g) of coffee samples as shown in the following equation: AC = (absorbance of sample–*y* − intercept) × DF/slope, where DF (dilution factor) = 50 *μ*l/ml; control curve equation *y* = −0.130*x* + 0.845*R*^2^ = 0.988.

While the antioxidant content was determined for all the coffee samples, the radical scavenging activity was checked only for the green and roasted extracts of Y1 (Y1g and Y1r) that showed better activity against all the test bacteria. The antioxidant test was done following Brand-Williams et al.'s method using DPPH as a free radical [16]. For determination of the DPPH IC50 percent inhibition, stock solutions of both the samples and the standard were prepared in six concentrations (66.677 *μ*g, 133.320 *μ*g, 266.640 *μ*g, 399.960 *μ*g, 533.280 *μ*g, and 666.670 *μ*g) dissolved in 1 ml 99% methanol. From each concentration, 1 ml volume mixed with 4 ml DPPH (40 *μg*/ml in 99% methanol). A blank was concurrently prepared by adding 1 ml of methanol to 4 ml DPPH of the above same concentration. All the samples, standard and the blank, were incubated in the dark for 30 minutes, and absorbance was read at 517 nm.

The test was performed three times in triplicate. Antioxidant activity (AA) or percent inhibition (% *I*) was expressed as a percentage of the DPPH radical inhibition after incubation with the test solutions with reference to the control sample and calculated from the equation AA (%*I*) = (Ac − As)/Ac × 100; where “Ac” and “As” are blank and sample absorbance, respectively.

### 2.6. Data Analysis

Data were analyzed using Minitab one-way ANOVA, and mean ± SD comparison was done by the post hoc Tukey test. *P* < 0.05 was considered statistically significant.

## 3. Results

### 3.1. Antibacterial Susceptibility

In a preliminary antibacterial test conducted before the actual experiment, cold-water extracts of both green and roasted coffee samples did not show any antibacterial activity. Similarly, instant and boiled coffee filtrates directly applied to well diffusion showed no activity. However, boiled coffee water, methanol, and ethyl acetate extracts showed antibacterial activity. The best inhibition zone was observed for boiled and instant coffee lyophilized products with slightly higher activity of the boiled than instant. The methanol extract showed better result following the boiled and instant coffee lyophilized water extracts. The ethyl acetate extract showed limited activity even against the otherwise most susceptible bacterial species (*S. aureus)* to the other extracts. Its activity was not even measurable against the rest of the bacteria. Further testing was done using boiled-lyophilized extracts based on the preliminary screening results.

All coffee samples demonstrated a significantly higher activity against all the test bacteria compared to the negative control (Table 1). There were, however, significant differences among the different coffee samples in their antibacterial activity. Bacterial susceptibilities were also significantly different for a particular sample. The third sample from Y (Y3) showed the highest activity against clinical *E. coli* (10.667 ± 0.577) with a significant difference from all other coffee samples. Against standard *E. coli*, similarly, the highest activity (12.667 ± 0.577) was found for Y3 next to the value for the positive control. Concerning the activity of the positive control against clinical and standard *E. coli* strains, it has better inhibitory activity against the standard than the clinical one.

Concerning *S. aureus*, on the other hand, Y1 showed the highest activity against both standard (23.333 ± 1.528) and clinical (22.667 ± 0.577) strains. These values were significantly higher than the measurements for the rest of the coffee samples. Here also, the activity of the positive control was better against the standard than the clinical strain.

Although all coffee samples effectively inhibited both clinical and standard strains of *S. typhimurium*, like against the other bacteria, the degree of inhibition was comparable with no statistically significant variation. Here again, the activity of chloramphenicol against the clinical bacterium was slightly lower than the activity against the standard even if the difference was negligible.

Clinical *P. aeruginosa* was best inhibited by Y1 (12.667 ± 1.528). Although the positive control showed activity at a higher concentration (1.5 mg/ml), it had no activity against this bacterium at the recommended test concentration. The inhibition zones of Y1, J1, and J3 against the standard strain of the bacterium were 13.667 ± 1.528, 13.333 ± 1.528, and 13.333 ± 1.528, respectively. Each of these records was significantly higher than all other treatments against the strain.

Overall, it appeared that Y1 was the most potent sample against six of the eight bacterial strains. S. *aureus* was the most susceptible for all the samples as well as the positive control and *E. coli* the least. *P. aeruginosa* was the most resistant to the test concentration of chloramphenicol with a nonmeasurable zone although some coffee extracts exhibited much better inhibitory activity against this bacterium. When the overall performance of the coffee samples of the two regions was compared, those from Y had relatively higher antibacterial activity than those from J concerning the clinical *E. coli* strain although there was no distinct pattern for others. Notwithstanding the significant difference between the samples from the three sources, the Ethiopian coffee generally has good antibacterial activity.

The lowest MIC (15.62 mg/ml) was against *S. aureus*, for both the standard and clinical strains. Against the rest of the bacteria, the value was 31.25 mg/ml. Similarly, the lowest MBC (31.25 mg/ml) was for *S. aureus* and for others, it was 62.50 mg/ml (Table 2).

### 3.2. Antioxidant Activity and Content

In the antioxidant activity test, the IC50 of green coffee (Y1g) was 167.426 *μ*g/ml (Table 3) whereas that of the roasted same type (Y1r) was higher (294.710 *μ*g/ml).

All coffee samples including those from the shop possessed considerable antioxidant content. This is true even for extracts at the lowest concentration. Nevertheless, all green coffee samples appeared to have slightly higher DPPH scavenging activity compared to their roasted counterparts. J3g had the lowest content compared to all other green coffee samples. Among the roasted samples, the highest was shown by Y3r whereas T the least (Table 4). The sample with the highest antibacterial activity (Y1) was also among those with the highest antioxidant activity.

## 4. Discussion

While the cold-water extract showed no antibacterial activity, the boiled one did well. The findings highlight that the Ethiopian traditional coffee ceremony bears some positive implications. In this long-standing tradition, coffee beans are manually roasted in an open-flame while carefully regulating the degree of roasting and immediately crushed using mortar and pistol, and soon brewed by boiling (decoction) using a specially designed pot (named *Jebena*) for this purpose. Our screening test suggested that boiling coffee added the antibacterial ingredients into the filtrate coffee beverage. In Ethiopia, a boiled coffee named *yejebenabunna* is the traditional way by which coffee is brewed mainly at home, and nowadays, the practice has widespread preference in public areas as well probably for its better stimulating effect and flavor. In this practice, coffee is boiled for an extended time (at least for about 15 minutes) together with the spent coffee grounds. Spent coffee residues exhibited antibacterial as well as antioxidant activities by other workers [17].

The relatively better activity of the positive control against nearly all of the standard strains than the clinical ones indicates chloramphenicol resistance. *P. aeruginosa* was least inhibited by the coffee extracts although the inhibition was significantly higher compared to the negative control, and surprisingly, it was the most resistant to the test concentration of chloramphenicol. The finding supports a previous study [18] that compared the antibacterial activity of Ethiopian coffee samples with those from Colombia and Kenya concluding that Ethiopian coffee was among the samples with the highest antimicrobial activity. That related study demonstrated that coffee had good inhibitory activity against *P. aeruginosa* in agreement with the current findings. *P. aeruginosa* is becoming a major health threat due to its resistance to most antibiotics available [19]. Selection of the best coffee type against this bacterium and identification of the active components may lead to the discovery of a lead compound(s) against the drug-resistant strains of this pathogen.

Variations between the extracts in their antibacterial activity may primarily be due to the genetic diversity of the coffee plants. Coffee samples from different countries showed antibacterial and antioxidant activity differences because of biochemical content difference, which in turn related to multiple limiting factors [20]. Ethiopia as the motherland of the coffee plant with diversified landscape and subsequent rainfall and temperature has diverse *C. arabica* cultivars. To this effect, the nation is the genetic baseline of the coffee plant [21]. As such, especially the southwestern Ethiopian coffee is the source of the coffee plants of other localities and coffee-owning nations at large [22]. Coffees of even single locality are of varied types, and the active ingredients differ accordingly. The Ethiopian cultivated coffee plants have varied fruit and bean morphology forming the basis to have about 13 coffee types. The coffee types or subtypes are of different aroma, test, pH, and other features.

Coffee plants grown within different microenvironments have different phenotypic and genotypic properties and, therefore, display different antibacterial activities. An investigation that compared the antimicrobial activity of one of the Ethiopian coffee types with those from other countries showed that the Ethiopian coffee had better or at least comparable activity. Apart from coffee plant phenotype and genotype, the sample type used, whether green or roasted, further affects the antibacterial and antioxidant activity of a coffee type, the roasting degree, field processing quality, laboratory extraction methods, solvents used, and brew making [11, 12]. The preliminary screening test of this study also showed the variation among these controlling factors on the antimicrobial activity.

The lowest MBC for *S. aureus* in this study shows the increased susceptibility of this bacterium to the coffee extracts. This study being on crude extracts has limitations, but if it were fractionated, the active ingredients that have specific activity would have been much better.

The green coffee sample had better antioxidant activity (IC50 = 167.426 *μ*g/ml) compared to the roasted coffee of the same sample type (IC50 = 294.710 *μ*g/ml) indicating that roasting somehow compromises the free radical scavenging activity of coffee. Nonetheless, all roasted and green coffee samples showed strong antioxidant activity. While studies elsewhere reported green coffee to have higher antioxidant activity compared to the roasted [20], others reported the reverse signifying that some coffee varieties have higher antioxidant activity in the roasted form while others in the green form [23]. Different factors may account for the relatively least antioxidant activity of the shop coffee in this study. The sample passed through different processing steps including blending and roasting until packaging and storage conditions (shelf life). This requires further investigation. The field samples, in contrast, are of high export quality (“specialty coffee”) and processed and tested immediately.

Factors that may contribute to the antioxidant activities of different coffee samples and thus to the apparent variations between findings include country of origin, degree of roasting, processing method, altitude at which the coffee plant was grown, soil type, average temperature, and number of sunny days per year, among others [20]. Roasting adds some antioxidant components like melanoidins due to Maillard reaction [24]. Contrarily, other highly antioxidant compounds are reported to be found in larger amount in green coffee but decreased in roasted coffee with increasing roasting degree. The roasting degree reported to have an effect on the antioxidant contents of coffee in that dark-roasted coffee showed the least antioxidant activity compared to medium and light. Green coffee showed the highest antioxidant activity indicating that the polyphenolic compounds that are very good antioxidants diminish with the increasing roasting degree [25, 26].

In general, coffee processing especially roasting affects the amount and availability of bioactive compounds known to have high antioxidant activity; as the roasting degree increases, phenolic compounds decrease. Even if roasting produces some compounds like melanoidins, they do not compensate the amount of highly bioactive phenolic compounds lost during roasting. Therefore, the recommendation is that to get the benefits of reducing diseases caused by oxidative stress through coffee, medium-to-light roasted coffee would be better [27].

The sample with the highest antibacterial activity (Y1) was also among those with the highest antioxidant content. This shows a correlation between antioxidant and antibacterial activities of our samples although other authors [28] did not find such correlation while working on other plants.

The results showed variability in the antioxidant content and activity of the coffee samples originating from the two regions and localities within each region. Future studies need to extend this work in greater depth as it seems that the agrogeography of the coffee plants varied determining coffee bean bioactive chemical content and type. Specific coffee-growing localities constitute specific coffee variants, and to select the best coffee type, more work is required. Oxidative reactions are becoming stresses to the world. Coffee has the highest antioxidant content compared to most other common beverages and is among the first beverages used everyday worldwide [20]. Therefore, more detailed study is required to derive the maximum possible benefit from the Ethiopian coffee.

A previous study on the antioxidant activity of a single sample from Y comparing it with 20 other Arabica coffee-producing countries showed its high activity. Yet, Y has diverse coffee-growing localities and coffee types with varied composition of bioactive components. As already stated, this investigation reports a variation in the bioactivity of the different locality coffee samples within a single coffee-growing region.

The preliminary phytochemical test showed the positivity of the coffee beans for the common bioactive compounds. There was higher amount of phenolic compounds. Among the most investigated polyphenols of coffee, chlorogenic acid is the dominant one especially in green coffee. This compound has numerous health benefits especially its antioxidant activity which is well-known. Caffeine is the other dominant bioactive component of coffee.

## 5. Conclusions

The coffee samples exhibited high antibacterial as well as antioxidant activities, although there was a variation with and within the region. A coffee sample with the most antibacterial activity was also among those with the highest antioxidant content. All the green coffee samples showed higher antioxidant content compared to roasted coffee samples regardless of origin. The antibacterial activities of the different coffee samples of the same region were variable, especially against the well-known drug-resistant *P. aeruginosa*. This variation suggests the variability in the composition of important bioactive compounds among specific coffee samples which is linked, among other factors, to a coffee plant variant and its growing locality. More in-depth investigation with larger sample size and different extraction solvents, roasting degree, and brewing process (decoction or infusion) is required to arrive at conclusive results.

## Figures and Tables

**Table 1:** Antibacterial activity of different coffee bean samples on different bacterial strains.

Sample	Ecc $\dot{X}\pm \text{SD}$	Staphc $\dot{X}\pm \text{SD}$	Salc $\dot{X}\pm \text{SD}$	Psedc $\dot{X}\pm \text{SD}$	Ecs $\dot{X}\pm \text{SD}$	Staphs $\dot{X}\pm \text{SD}$	Sals $\dot{X}\pm \text{SD}$	Pseds $\dot{X}\pm \text{SD}$
Y1	9.333 ± 0.577^a^	22.667 ± 0.577^a^	17.333 ± 0.557^a^	12.667 ± 1.528^a^	10.000 ± 0.000^a^	23.333 ± 1.582^a^	18.333 ± 1.155^a^	13.667 ± 1.528^a^
Y2	8.333 ± 0.577^b^	20.667 ± 0.577^b^	17.667 ± 0.577^a^	8.667 ± 1.000^b^	8.333 ± 0.577^b^	20.667 ± 1.155^b^	18.000 ± 1.000^a^	9.000 ± 1.000^b^
Y3	10.667 ± 0.577^c^	19.333 ± 1.528^c^	17.667 ± 0.577^a^	11.667 ± 1.155^c^	12.667 ± 0.577^c^	20.333 ± 1.528^b^	17.667 ± 0.577^a^	11.667 ± 1.528^c^
J1	8.333 ± 0.577^b^	21.000 ± 1.000^d^	17.333 ± 0.577^a^	12.000 ± 1.000^a^	8.667 ± 0.577^b^	23.333 ± 1.155^c^	18.333 ± 0.577^a^	13.333 ± 1.528^a^
J2	8.667 ± 0.577^b^	20.333 ± 0.577^b^	17.667 ± 0.000^a^	9.667 ± 0.577^d^	9.000 ± 1.000^b^	20.667 ± 0.577^b^	17.667 ± 0.577^a^	9.667 ± 0.577^b^
J3	8.333 ± 0.577^b^	19.333 ± 0.577^c^	17.000 ± 0.000^a^	11.667 ± 1.528^e^	9.000 ± 1.000^b^	21.000 ± 1.000^b^	18.000 ± 0.000^a^	13.333 ± 1.528^a^
T	8.667 ± 0.577^b^	21.667 ± 0.577^d^	16.667 ± 0.577^a^	10.000 ± 1.000^f^	9.333 ± 0.577^b^	20.000 ± 1.000^b^	17.333 ± 0.577^a^	11.667 ± 0.577^c^
Chl	12.333 ± 0.577^d^	24.333 ± 0.577^e^	23.000 ± 1.000^b^	8.000 ± 0.000^b^	13.333 ± 0.577^d^	25.667 ± 0.577^d^	23.667 ± 0.577^b^	9.000 ± 0.000^b^
DW	0.000 ± 0.000^e^	0.000 ± 0.000^f^	0.000 ± 0.000^c^	0.000 ± 0.000^g^	0.000 ± 0.000^e^	0.000 ± 0.000^e^	0.000 ± 0.000c	0.000 ± 0.000^d^

Statistical differences were analyzed by ANOVA (*P* < 0.05). Different letters indicate significant differences between the samples on each bacterium (Tukey test pairwise comparison). *X*: mean diameter of inhibition zone (mm) including a diameter of 6 mm well. Ecc: clinical *E. coli*; Staphc: clinical *S. aureus*; Staphs: standard *S. aureus*; Salc: clinical *Salmonella typhimurium*; Psedc: clinical *P. aeruginosa*; Ecs: standard *E. coli*; Sals: standard *Salmonella typhimurium*; Pseds: standard *P. aeruginosa*; J: Jimma; Y: Yirgacheffe; Chl.: chloramphenicol (positive control); DW: distilled water (negative control), T: shop coffee; SD: standard deviation.

**Table 2:** MIC and MBC of Y1r against different bacterial strains.

Bacteria	MIC (mg/ml)	MBC (mg/ml)
Ecc	31.25	62.50
Ecs	31.25	62.50
Stc	15.62	31.25
Sts	15.62	31.25
Sac	31.25	62.50
Sas	31.25	62.50
Pac	31.25	62.50
Pas	31.25	62.50

Ecc: *E. coli* clinical; Ecs: *E. coli* standard; Stc: *S. typhimurium* clinical; Sts: *S. typhimurium* standard; Sac: *S. aureus* clinical; Sas: *S. aureus* standard; Pac: *P. aeruginosa* clinical; Pas: *P. aeruginosa* standard; MIC: minimum inhibitory concentration; Y1r: Yirgacheffe sample 1 roasted.

**Table 3:** DPPH assay results for Y1g and Y1r taking ascorbic acid as a positive control.

Concentration (*μ*g/ml)	% *I*			IC50	
	Y1g	Y1r	Aa	Y1g	Y1r
66.677	35.544	23.402	90.886		
133.320	45.690	31.931	90.506		
266.640	56.887	42.605	92.110	167.426	
399.960	88.805	58.579	92.110		294.710
533.280	89.929	90.059	92.490		
666.670	91.432	90.342	92.575		

IC50: 50% inhibitory concentration; % *I*: percentage inhibition; Aa: ascorbic acid; Y1g: Yirgacheffe 1 green; Y1r: Yirgacheffe 1 roasted.

**Table 4:** Antioxidant contents of coffee samples using ascorbic as a standard and DPPH as a free radical.

Sample	AEAC (*μ*g/ml)
J1g	311.160
J1r	304.754
J2g	311.019
J2r	305.807
J3g	308.076
J3r	302.000
Y1g	310.485
Y1r	304.303
Y2g	310.184
Y2r	300.092
Y3g	311.596
Y3r	307.948
T	293.215

AEAC: ascorbic acid equivalent antioxidant content; J1g: Jimma 1 green; J1r: Jimma 1 roasted; J2g: Jimma 2 green; J2r: Jimma 2 roasted; J3g: Jimma 3 green; J3r: Jimma 3 roasted; Y1g: Yirgacheffe 1 green; Y1r: Yirgacheffe 1 roasted; Y2g: Yirgacheffe 2 green; Y2r: Yirgacheffe 2 roasted; Y3g: Yirgacheffe 3green; Y3r: Yirgacheffe 3 roasted; T: shop coffee.

## Data Availability

The data used to support the findings of this study are included within the article.
